# Primary leiomyosarcoma of thyroid with pulmonary metastasis: A diagnostic odyssey

**DOI:** 10.1002/ccr3.8875

**Published:** 2024-05-10

**Authors:** Safi Ullah, Hamza Fazal, Subtain Hassan, Muhammad Saqib, Abdul Wali Khan, Abdulqadir J. Nashwan, Irfan Ullah

**Affiliations:** ^1^ Kuwait Teaching Hospital Peshawar Pakistan; ^2^ Department of Internal Medicine Khyber Teaching Hospital Peshawar Pakistan; ^3^ University of Missouri Kansas City‐School of Medicine Kansas Montana USA; ^4^ Hamad Medical Corporation Doha Qatar; ^5^ Kabir Medical College Gandhara University Peshawar Pakistan; ^6^ Institute of Public Health and Social Sciences (IPH&SS) Khyber Medical University Peshawar Pakistan

**Keywords:** dyspnea, leiomyosarcoma, lymphadenopathy, male, thyroid gland

## Abstract

The presented primary thyroid leiomyosarcoma (TL) case report underscores the importance of recognizing and addressing the diagnostic challenges and management complexities associated with this exceedingly rare malignancy. Given the limited effective therapeutic strategies available, timely intervention, thorough diagnostics, and vigilant follow‐up are paramount in managing such intricate tumors. Further research focusing on molecular‐based treatment modalities is imperative to improve patient outcomes in cases of primary TL.

## Graphical abstract

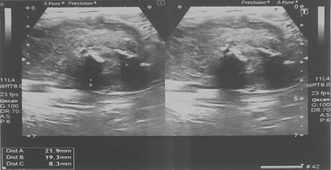

## 1 INTRODUCTION

Thyroid cancers are among the most prevalent neoplasms, primarily affecting women. Ninety to ninety‐five percent of cases of differentiated thyroid carcinomas are follicular and papillary carcinomas.^1^ Of all thyroid cancers, medullary thyroid carcinoma accounts for 6% of cases, whereas papillary carcinoma is the most common type.^1^ Sarcomas, lymphomas, and anaplastic carcinomas are among the less common forms. Sarcoma is a very uncommon type of tumor. Angiosarcoma, leiomyosarcoma (LMS), and liposarcoma are the three sarcoma types that have been seen in the thyroid glands.^1^ Primary thyroid leiomyosarcoma (TL) is a rare malignant tumor of mesenchymal origin, primarily emerging from the smooth muscle cells within the thyroid capsule's vessels. Within the realm of primary thyroid cancers, it comprises a mere 0.014%, characterized by its highly aggressive clinical trajectory and, thus, a markedly dismal survival rate.^2^ The World Health Organization (WHO) classification for thyroid and parathyroid tumors delineates smooth muscle tumors into two categories: benign (leiomyoma) and malignant (leiomyosarcoma).^3^

The head and neck region encompasses around 20% of sarcoma cases, affecting predominantly the oral cavity, subcutaneous soft tissues, sinus linings, and, sporadically, the thyroid gland. In the domain of leiomyosarcomas, their onset typically manifests in the elderly population, with the average age of reported cases being 66 years. Notably, gender holds no sway over its occurrence.^4^ Among documented cases, more than half have chronicled the emergence of distant metastases, with the lungs and liver being the primary sites of involvement.^5^

Given the confinement of the tumor within the thyroid, the recommended surgical intervention entails a total thyroidectomy coupled with lymph node dissection. However, in instances where the neoplasm infiltrates neighboring structures, resorting to surgical modalities alongside radiotherapy and/or chemotherapy has exhibited minimal success in enhancing the prognosis. Distinguishing TL from anaplastic thyroid carcinoma poses a diagnostic challenge due to the complexities of achieving preoperative differentiation.^6^ This case underscores the rarity of primary TL and the challenges in preoperative diagnosis due to its indistinct imaging features.

## 2 CASE PRESENTATION

A 66‐year‐old male presented to the Accident and Emergency Department of a teaching hospital with a constellation of distressing symptoms. He reported experiencing dysphagia and dyspnea associated with the ingestion of solid foods and liquids. In addition, the patient described a gradually enlarging mass in his neck, which he had noticed a few months prior. Notably, the patient's health had been relatively uneventful until the sudden onset of these symptoms. The patient's medical history was devoid of any prior significant medical conditions. There was no history of alcohol consumption or esophageal strictures. Upon physical examination, the patient presented with stable vital signs, although palmar sweating and fine tremors in his hands during extension were noted. When asked to lie down, the patient exhibited an onset of shortness of breath. A neck examination revealed the presence of a well‐defined, 6–8 cm solid mass in the right anterior cervical region, affixed to both superficial and deep tissue layers. The thyroid gland appeared to be functioning normally, and thyroid autoantibodies were undetectable.

## 3 METHODS

**FIGURE 1:**
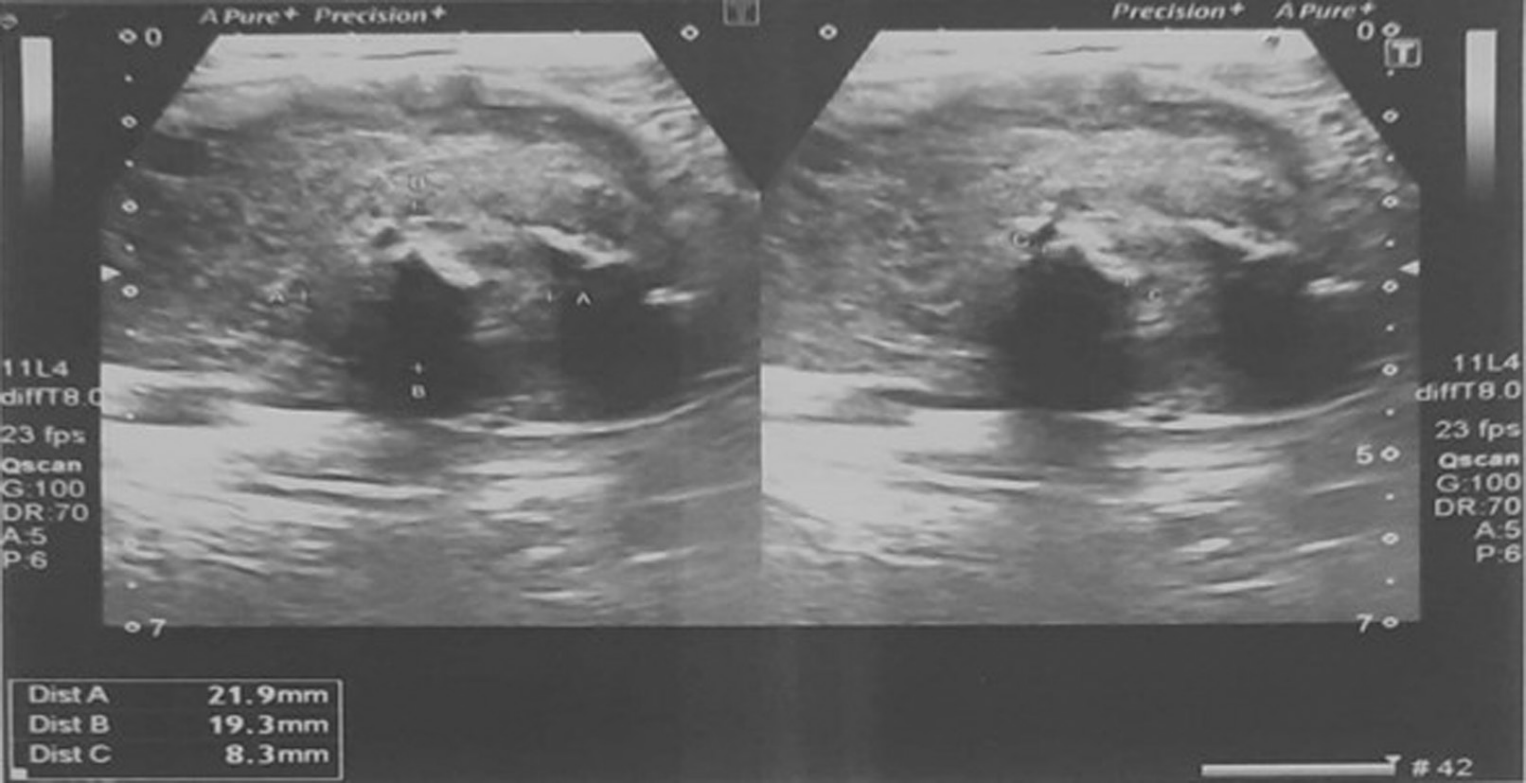
Ultrasound thyroid of a patient with primary leiomyosarcoma of the thyroid.

**FIGURE 2:**
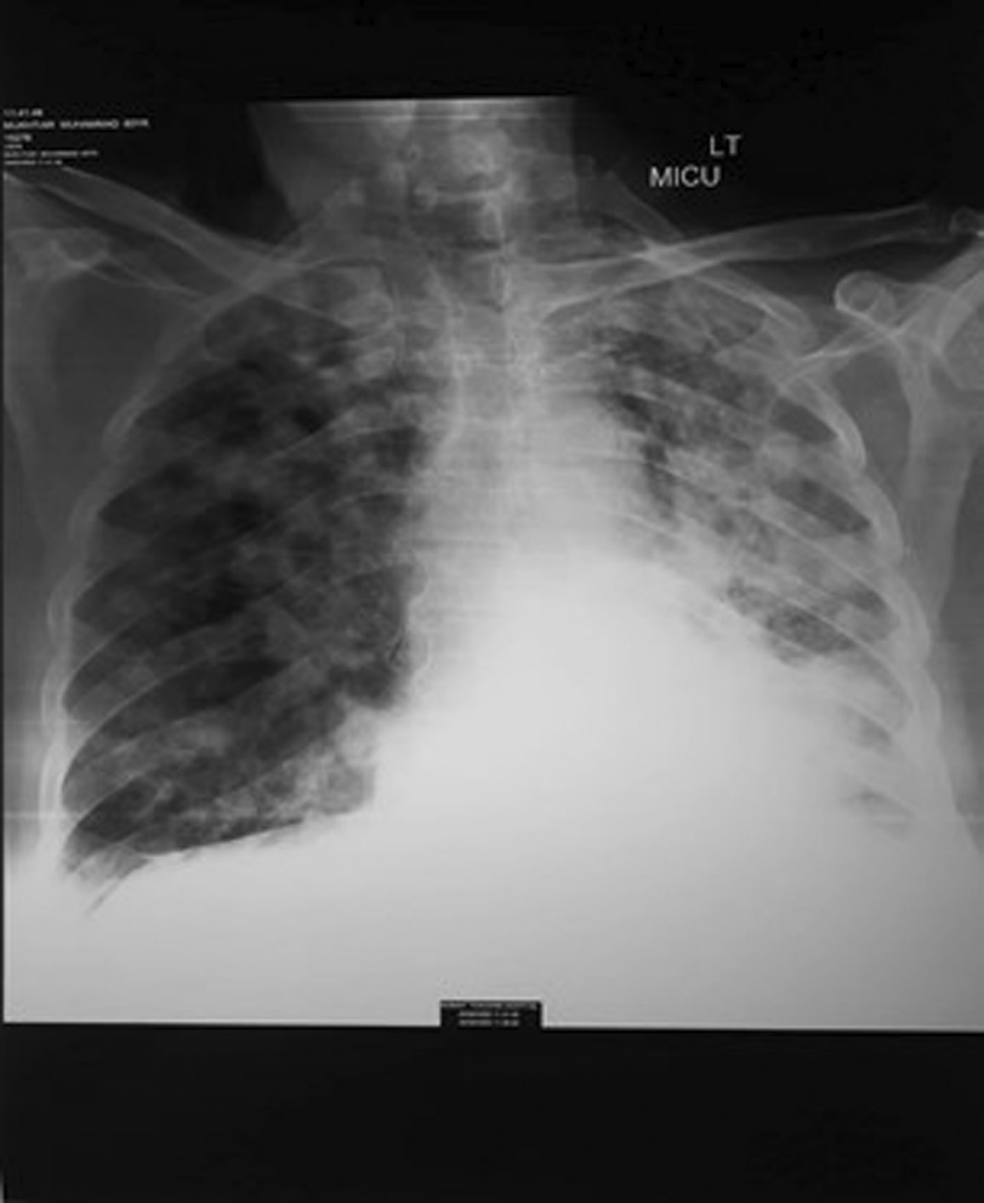
Chest x‐ray with metastasis from primary leiomyosarcoma of the thyroid.

Thyroid ultrasonography displayed a heterogeneous, hypodense mass (measuring 60 × 39 × 33 mm) within the right thyroid lobe, exhibiting both peri‐ and intralesional vascular flow (Figure 1). Fine needle aspiration cytology confirmed the diagnosis of undifferentiated malignancy. A Chest x‐ray shows metastasis, as shown in (Figure 2). The patient was referred for a total thyroidectomy, which subsequently underwent histopathological and immunohistochemical analysis. The evaluation revealed a leiomyosarcoma, FNCLCC grade‐3, measuring 7.8 cm in diameter. The tumor displayed marked atypia and focal areas of necrosis. Notably, the left thyroid lobe exhibited benign tissue. Immunohistochemical staining demonstrated the absence of CK, TTF1, PAX8, Desmin, and S‐100 reactivity, whereas SMA was positive. Given these findings, Leiomyosarcoma was favored over sarcomatoid carcinoma as the diagnosis.

**FIGURE 3:**
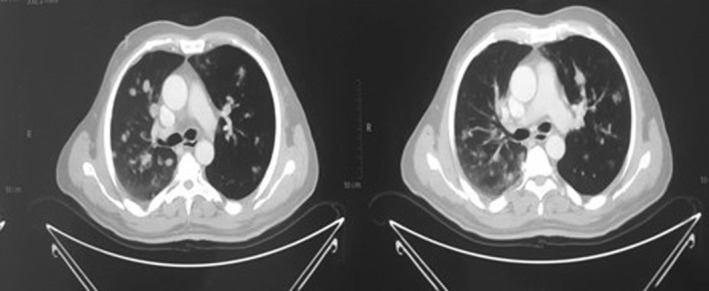
CT chest with metastasis from primary leiomyosarcoma of thyroid. Metastasis mark red.

Subsequent CT scans of the chest uncovered post‐surgical changes in the thyroid bed and highlighted pulmonary nodules, raising concerns of metastasis (Figure 3).

MRI of the brain with contrast revealed no enhancing brain parenchymal lesions or leptomeningeal involvement. The ventricular system and posterior fossa structures were within normal limits. The pituitary gland was not enlarged, and no significant abnormalities were noted.

A PET‐CT scan of the head and neck exposed hypermetabolic enlargement of the right upper cervical lymph nodes at levels II and III. These nodes measured 2.5 × 1.8 cm and 1.7 cm, respectively, and exhibited elevated standardized uptake value (SUV). Furthermore, multiple hypermetabolic pulmonary nodules were identified bilaterally within the lungs, indicative of metastatic involvement. Various pulmonary nodules with distinct SUV values were observed, suggesting metastatic spread. Additionally, thrombi in the right internal jugular vein and a filling defect in the lobar branch of the right upper lobe pulmonary artery were detected.

## 4 RESULTS

The patient was then referred for chemotherapy, five sessions of chemotherapy (Gemcitabine and Docetaxel). Later, he presented in the emergency with shortness of breath due to mets in his lung and was admitted to ICU; after recovery, he was discharged and referred to an oncologist, who put on oral chemotherapy. He was fine for 2 months, again presented in Emergency with the same complaint and expired.

In summation, this comprehensive case presentation details the clinical journey of a 66‐year‐old male who presented with dysphagia, dyspnea, and a growing neck mass. A thorough investigation revealed a leiomyosarcoma originating from the thyroid, with subsequent metastatic spread to the lungs. The case underscores the importance of timely intervention, comprehensive diagnostics, and diligent follow‐up in managing complex malignancies.

## 5 DISCUSSION

Leiomyosarcomas are commonly found in the gynecological and gastrointestinal systems, as well as in the retroperitoneum. While this tumor type constitutes approximately 4% of head and neck sarcomas, primary TL remains an exceedingly rare occurrence.^7^ However, advancements in diagnostic immunohistochemical methods have contributed to an increasing number of reported cases in recent times.^7^ This study introduces an additional case to the existing literature.

Interestingly, this tumor appears to exhibit a slight predilection for female patients, and the mean age at diagnosis, inclusive of our presented case, is 63.4 years, with an age range spanning from 32 to 90 years. Moreover, a unique instance of Epstein–Barr virus‐associated primary TL surfaced in a 6‐year‐old patient with congenital immunodeficiency.^8^

Among the common patient complaints, the rapid growth of a neck mass prevails, aligning with our observations. Additional symptoms encompass dysphagia (as evidenced in our case), hoarseness, weight loss, dyspnea, and arm pain.^9^

Rendering a preoperative diagnosis for primary TL can be notably challenging. The tumor lacks characteristic imaging features that would be instrumental for diagnostic purposes. Noteworthy differentials in pathological diagnosis encompass anaplastic thyroid carcinoma, a spindle cell variant of medullary carcinoma, solitary fibrous tumor, and spindle epithelial tumor with thymus‐like differentiation (SETTLE).^10^ Fédération Nationale des Centres de Lutte Contre le Cancer (FNCLCC) grading for soft tissue is on the basis of the sum of total tumor differentiation, mitotic count, and tumor necrosis scores. FNCLCC classifies tumors into grade 1 (2–3 points), grade 2 (4–5 points), and grade 3 (6–8 points).^11^

Our experience, much like other documented cases, reveals that the diagnosis of primary TL is typically established post‐surgery, hinging on the tumor's immunohistochemical attributes. In the majority of cases, patients present as euthyroid. Ultrasonography may unveil an ill‐defined or well‐defined hypoechoic mass, occasionally accompanied by calcified or cystic components. Thyroid isotope scanning has the capacity to disclose a cold nodule or hyperplasia characterized by varying radioactive iodine uptake. Despite its utility, fine needle aspiration cytology often falls short in differentiating the rare primary TL from the more prevalent anaplastic carcinomas. This distinction is complicated by the occasional morphological resemblance of anaplastic carcinomas to sarcomas.^10^

Microscopically, neoplastic cells exhibit elongation, featuring abundant acidophilic fibrillary cytoplasm and a nucleus typically positioned centrally, displaying a blunt‐ended or “cigar‐shaped” appearance. The extent of nuclear atypia exhibits substantial variability, and mitotic activity similarly demonstrates considerable variation. While a high mitotic index significantly points toward malignancy, suspicion of primary TL should be retained for tumors exhibiting extensive necrosis, hemorrhage, and pronounced atypia, even in cases with low mitotic activity.^12^ Immunohistochemical stains for keratins, detected in 50–100% of cases, corroborate the epithelial nature of the tumor.

As of now, the efficacy of therapeutic interventions in prolonging survival remains ambiguous, as indicated by the analysis of 19 reported cases.^13^ Total or near‐total thyroidectomy, coupled with therapeutic modified radical neck dissection, is advisable for intrathyroidal disease, a strategy employed for the majority of thyroid pathologies.^14^ Frequently, therapies yield minimal clinical benefits, largely resulting in palliative outcomes.

## 6 CONCLUSION

Primary TL continues to present as a formidable tumor, consistently tied to an unfavorable prognosis. Despite advancements in the field of oncology, the absence of a comprehensive and effective multimodal treatment regimen persists. Addressing the challenges posed by suboptimal surgical outcomes necessitates the development of innovative adjuvant therapeutic strategies rooted in molecular insights. The case report underscores the urgent need for further research and clinical efforts to better understand and combat this rare and aggressive malignancy.

## AUTHOR CONTRIBUTIONS

**Safi Ullah:** Writing – original draft; writing – review and editing. **Hamza Fazal:** Writing – original draft; writing – review and editing. **Subtain Hassan:** Writing – original draft; writing – review and editing. **Muhammad Saqib:** Writing – original draft; writing – review and editing. **Abdul Wali Khan:** Writing – original draft; writing – review and editing. **Abdulqadir J. Nashwan:** Writing – original draft; writing – review and editing. **Irfan Ullah:** Writing – original draft; writing – review and editing.

## FUNDING INFORMATION

Open access fund provided by the Qatar National Library.

## CONFLICT OF INTEREST STATEMENT

The authors have no conflicts of interest to declare.

## ETHICS STATEMENT

Ethical approval is not required for this study in accordance with local or national guidelines.

## CONSENT

Written informed consent was obtained from the patient to publish this report in accordance with the journal's patient consent policy.

## Data Availability

All data generated or analyzed during this study are included in this article. Further inquiries can be directed to the corresponding author.
